# Fish Invasions in the World's River Systems: When Natural Processes Are Blurred by Human Activities 

**DOI:** 10.1371/journal.pbio.0060028

**Published:** 2008-02-05

**Authors:** Fabien Leprieur, Olivier Beauchard, Simon Blanchet, Thierry Oberdorff, Sébastien Brosse

**Affiliations:** 1 Laboratoire Evolution and Diversité Biologique, UMR 5174, CNRS-Université Paul Sabatier, Toulouse, France; 2 Faculty of Sciences, Department of Biology, Ecosystem Management Research Group University of Antwerp, Antwerpen (Wilrijk), Belgium; 3 Département de Biologie, Centre Interuniversitaire de Recherche sur le Saumon Atlantique (CIRSA) and Québec-Océan, Université Laval, Sainte-Foy, Quebec City, Quebec, Canada; 4 Institut de Recherche pour le Développement (UR131), Antenne au Muséum National d'Histoire Naturelle, Paris, France; University of Tennessee, United States of America

## Abstract

Because species invasions are a principal driver of the human-induced biodiversity crisis, the identification of the major determinants of global invasions is a prerequisite for adopting sound conservation policies. Three major hypotheses, which are not necessarily mutually exclusive, have been proposed to explain the establishment of non-native species: the “human activity” hypothesis, which argues that human activities facilitate the establishment of non-native species by disturbing natural landscapes and by increasing propagule pressure; the “biotic resistance” hypothesis, predicting that species-rich communities will readily impede the establishment of non-native species; and the “biotic acceptance” hypothesis, predicting that environmentally suitable habitats for native species are also suitable for non-native species. We tested these hypotheses and report here a global map of fish invasions (i.e., the number of non-native fish species established per river basin) using an original worldwide dataset of freshwater fish occurrences, environmental variables, and human activity indicators for 1,055 river basins covering more than 80% of Earth's surface. First, we identified six major invasion hotspots where non-native species represent more than a quarter of the total number of species. According to the World Conservation Union, these areas are also characterised by the highest proportion of threatened fish species. Second, we show that the human activity indicators account for most of the global variation in non-native species richness, which is highly consistent with the “human activity” hypothesis. In contrast, our results do not provide support for either the “biotic acceptance” or the “biotic resistance” hypothesis. We show that the biogeography of fish invasions matches the geography of human impact at the global scale, which means that natural processes are blurred by human activities in driving fish invasions in the world's river systems. In view of our findings, we fear massive invasions in developing countries with a growing economy as already experienced in developed countries. Anticipating such potential biodiversity threats should therefore be a priority.

## Author Summary

As one of the major threats to biodiversity, the detrimental consequences of biological invasions are widely recognised. Despite this, a global view of invasion patterns and their determinants is still lacking in aquatic ecosystems, reducing our ability to initiate practical actions. Here we report the global patterns of freshwater fish invasion in 1,055 river basins covering more than 80% of Earth's continental surface. This allows us to identify six major invasion hotspots where non-native species represent more than a quarter of the total number of species. According to the World Conservation Union, these areas are also characterised by the highest proportion of threatened fish species. We also show that the natural factors controlling global biodiversity do not influence the number of non-native species in a given river basin. Instead, human activity–related factors, and particularly economic activity, explain why some river basins host more non-native species. In view of our findings, we fear massive invasions in developing countries with a growing economy as already experienced in developed countries. This constitutes a serious threat to global biodiversity.

## Abstract 3

Mapping worldwide freshwater fish invasions allowed the identification of major invasion hot spots and demonstrated that economic activity is the main determinant of freshwater fish invasions at the global scale.

## Introduction

The deliberate or accidental introduction of species outside their native range is a key component of the human-induced biodiversity crisis, harming native species and disturbing ecosystems processes [1–3]. The greater the introduction of non-natives in a region, the higher the probability that some of them become invasive and will hence cause ecological or economic damage [4,5]. Patterns of non-native species richness are therefore relevant in forecasting the overall impact of invasions on a global scale [5] and should help management authorities to adopt sound, effective conservation policies [5–7].

The process of species invasion consists of three successive stages: initial dispersal, establishment of self-sustaining populations, and spread into the recipient habitat. The last two stages are contingent upon the first one, i.e., if initial dispersal is interrupted, establishment and spread do not occur [8]. Three major hypotheses, which are not necessarily mutually exclusive, have been proposed to explain invasion patterns: the “human activity” [9], “biotic acceptance” [10], and “biotic resistance” [11] hypotheses. The “human activity” hypothesis refers to the three stages of the invasion process (initial dispersal, establishment, and spread), whereas the “biotic resistance” and “biotic acceptance” hypotheses address only the establishment and spread stages [12]. With regards to the establishment stage, the “human activity” hypothesis predicts that, by disturbing natural landscapes and increasing propagule pressure (i.e., the number of individuals released and the frequency of introductions in a given habitat), human activities facilitate the establishment of non-native species [9,13,14]. Everything else being equal, a positive relationship is therefore expected between non-native species richness and quantitative surrogates of propagule pressure and habitat disturbance (e.g., gross domestic product [GDP], percentage of urban area, and human population density [5]). Then, the “biotic acceptance” hypothesis predicts that the establishment of non-native species would be greatest in areas that are rich in native species and with optimal environmental conditions for growth (i.e., “what is good for natives is good for non-natives too” [10]). Everything else being equal, native and non-native species richness should co-vary positively with environmental factors such as energy availability and habitat heterogeneity, which are already recognised as the primary global determinants of native species richness [15,16]. In contrast, the “biotic resistance” hypothesis predicts that species-poor communities will host more non-native species than species-rich communities, the latter being highly competitive and hence readily impede the establishment of non-native species [11,17]. Therefore, a negative relationship is expected between native and non-native species richness. To date, the relative importance of these hypotheses in explaining the variation in non-native species richness had never been tested at the global scale.

We tested these hypotheses and report a global map of fish invasions (i.e., the number of non-native fish established per river basin) by using an extensive worldwide dataset of freshwater fish occurrences (i.e., more than 40,000 occurrences of 9,968 fish species) on the river basin scale (1,055 basins covering more than 80% of Earth's surface). Freshwater fish offer a unique opportunity to identify factors that are responsible for large-scale gradients in non-native species richness for at least two main reasons. First, among vertebrate groups, freshwater fish have been widely introduced over the world [18], which often had subsequent negative consequences on native species and ecosystems integrity [19–23]. Second, as rivers are separated from one another by barriers insurmountable for freshwater fish (land or ocean), they form kind of “biogeographical islands”, whose space is delimited [15]. This implies that the natural and human factors shaping global patterns of non-native species richness can be easily separated.

## Results

**Figure 1:**
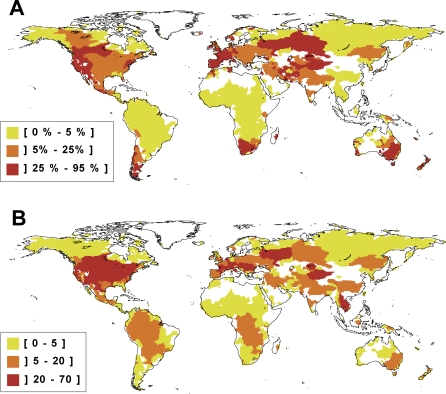
Worldwide Distribution of Non-Native Freshwater Fish (A) The percentage of non-native species per basin (i.e., the ratio of non-native species richness/total species richness) and (B) the non-native species richness per basin. Each basin was delimited by a GIS using 0.5° × 0.5° unit grid. The maps were drawn using species occurrence data for 9,968 species in 1,055 river basins covering more than 80% of continental areas worldwide. Invasion hotspots are defined as areas where more than a quarter of the species are non-native (red areas on map (A)), leading to define six invasion hotspots: the Pacific coast of North and Central America, southern South America, western and southern Europe, central Eurasia, South Africa and Madagascar, southern Australia, and New Zealand.

**Figure 2:**
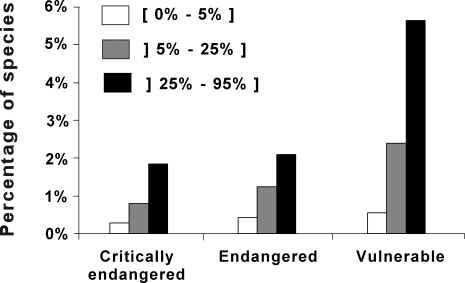
Percentage of Threatened Species for the Three Invasion Levels Threatened species were identified from the IUCN Red List (vulnerable, endangered, critically endangered). We calculated the percentage of threatened species, listed in the IUCN Red List, for the three invasion levels considered in Figure 1A. Each invasion level expessed as the percentage of non-native species. ([ 0%–5% ], ]5%–25%], ]25%–95%]) account for 8,363, 2,257, 1,241 native species and 544, 240, 271 river basins, respectively.

Our results revealed six global invasion hotspots where non-native species represent more than a quarter of the total number of species per basin: the Pacific coast of North and Central America, southern South America, western and southern Europe, Central Eurasia, South Africa and Madagascar, and southern Australia and New Zealand (Figure 1A). According to The World Conservation Union (IUCN) Red List [25], these areas were also characterised by the highest proportion of fish species having a high risk of extinction in the wild (Figure 2).

Analysing the absolute number of species, we found that river basins of the Northern Hemisphere host the highest number of non-native fish species (Figure 1B). The human factors considered here to test the “human activity” hypothesis (GDP, population density, percentage of urban area) were found to be positively related to non-native species richness (Table 1), after controlling for the effects of environmental conditions and native species richness. In contrast, the positive correlation between native and non-native richness that was expected by the “biotic acceptance” hypothesis was not significant after controlling for the effects of propagule pressure and habitat disturbance (Table 1). Indeed, the environmental factors displayed either no (net primary productivity) or a weak positive correlation (altitudinal range and basin area) with non-native species richness, after controlling for the effects of propagule pressure and habitat disturbance (Table 1). The negative correlation between native and non-native richness, expected by the “biotic resistance” hypothesis, was not significant after controlling for the effects of environmental conditions, propagule pressure, and habitat disturbance (Table 1).

**Table 1:**
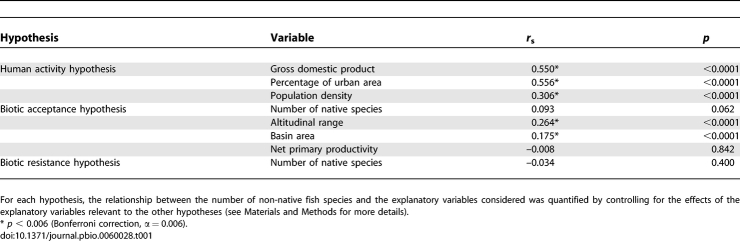
Spearman Rank Correlation (*r*_s_) between the Number of Non-Native Fish Species (Residuals) and Each Explanatory Variable Related to the “Human Activity,” “Biotic Acceptance,” and “Biotic Resistance” Hypotheses (*n* = 597)

Then, we applied hierarchical partitioning [26–28] that aims to quantify the independent explanatory power of each variable by considering all possible submodels. The deviance explained by the 128 submodels computed in hierarchical partitioning accounted in average for 52% of the total deviance (±7% standard deviation [SD], min = 37%, max = 67%). The human factors had together the greatest independent effect on non-native species richness (70%, Table 2). Among the human factors, the GDP (an economical index of human activities [9]) had the greatest independent explanatory power (43%; Table 2). To a lesser extent, the habitat heterogeneity (i.e., basin area and altitudinal range) and the number of native species also contribute to the variation in non-native species between river basins (Table 2).

**Table 2:**
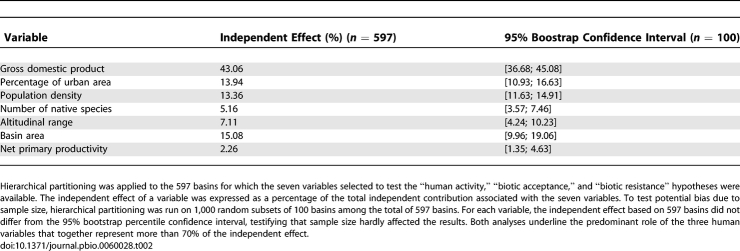
Independent Effect of Each Environmental and Human Activity–Related Variable on the Number of Non-Native Species per Basin

To test for potential bias in our results due to differences in sampling effort between continents, bootstrap analysis was performed by applying hierarchical partitioning to 1,000 random subsets of 100 basins. For each variable, the independent effect observed did not differ from the 95% bootstrap percentile confidence interval (Table 2), testifying that potential differences in sampling effort between continents hardly affected the results.

## Discussion

By using an explanatory modelling approach, we showed that the human activity indicators of the world's river basins were positively related to the number of established non-native fish species. In addition, they account for most of the global variation in non-native species richness, giving support for the “human activity” hypothesis. More particularly, we highlight that the level of economic activity of a given river basin (expressed by the GDP) strongly determines its invasibility. Three non-exclusive mechanisms may account for this pattern. First, economically rich areas are more prone to habitat disturbances (e.g., dams and reservoirs modifying river flows) that are known to facilitate the establishment of non-native species [7,23,29]. Second, high rates of economic exchanges increase the propagule fluxes of non-native species [6,9] via ornamental trade, sport fishing, and aquaculture [18]. Third, the increased demand for imported products associated with economic development increase the likelihood of unintentional introductions through the import process [6].

The “biotic resistance” hypothesis cannot explain the pattern of fish invasions observed, because no negative relationship between native and non-native species richness was found after controlling for the effects of environmental conditions, propagule pressure, and habitat disturbance. This means that regional species-rich communities are not necessarily a barrier against the establishment of non-native species [17]. Our results are consistent with several studies showing that species-rich fish communities can support higher species richness if the pool of potential colonisers is increased by species introductions [24,30,31]. More generally, our results agree with studies on various taxa that do not report biotic resistance at broad spatial scales [10,11]. Then, we provide no real support for the alternative “biotic acceptance” hypothesis [10] even if native and non-native species richness do respond similarly to some of the environmental gradients tested (i.e., altitudinal range and basin area). Actually, the absence of a significant positive relationship between native and non-native species richness implies that species-rich river basins do not support more non-native species than basins with a low native species richness (i.e., “the rich do not get richer”). This contrasts with numerous continental and regional-scale studies on plants and animals that report a strong matching between native and non-native species richness [10,32–35]. More generally, our results do not agree with the expectation that native and non-native species richness covary positively at macroecological scales [36].

The interpretation of the exact role of human activities (i.e., propagule pressure and habitat disturbance) in driving broad-scale patterns of non-native species richness faced major difficulties in previous continental and regional-scale studies due to covariations between human and natural factors [9,13,34,35]. Indeed, because humans may have preferred to settle in areas providing diverse natural resources, human population was found to be largest in regions with high levels of habitat heterogeneity and energy availability that favour species-rich native fauna and flora [34,37]. This therefore makes it difficult to determine whether the often-reported positive relationship between native and non-native species richness is driven by (i) common responses to habitat heterogeneity and energy availability or (ii) increased propagule pressure and habitat disturbance. Such difficulties were probably related to the spatial extent considered (i.e., a continental or regional extent). Indeed, we found a weak covariation between environmental and human descriptors of the world's river basins at the global scale (Pearson's correlation coefficients: *r* < 0.35, Table S1). This allowed us to clearly disentangle the relative roles of human activities and environmental conditions in shaping the global pattern of fish invasions. We show that the biogeography of fish invasions at the global scale matches the geography of human impact but not the biogeography of native species.

Because increasing the number of non-native species increases the risk of biodiversity loss [4,5], our results have two major implications for future conservation strategies. First, the six global invasion hotspots identified here account for the highest proportion of threatened fish species listed on the IUCN Red List [25]. These areas are also recognised as being biodiversity hotspots (particularly southern Europe, South Africa and Madagascar, southern Australia, and New Zealand [38,39]). Although species classified on the IUCN Red List are threatened by various sources of disturbance (e.g., habitat loss, pollution, species invasion, and overexploitation [25]), non-native species are recognised as a major threat to biodiversity after habitat loss [25,40]. For example, 20% of the 680 species extinctions listed by the IUCN were directly caused by species invasions [2]. Freshwater fish follow the same tendency, as 20% of the species listed by the IUCN are threatened by non-native species [41]. In that context, we recommend that non-native species importations in the six invasion hotspots be prohibited without detailed risk and long term cost-benefits assessments [42]. Special attention should also be given to these areas to design efficient control programs of already-established non-native species.

Second, as we provide strong evidence for the “human activity” hypothesis (with a special emphasis on economic activity), we expect that river basins of developing countries will host an increasing number of non-native fish species as a direct result of economic development. This constitutes a serious threat to global biodiversity, because rivers of most developing areas (e.g., southern Asia, western and central Africa) are characterised by high levels of endemism [38]. Anticipating potential biodiversity threats should therefore be a priority, because once they are established, the eradication of a non-native species is extremely difficult and result in high economic costs [43].

Despite the increasing literature on non-native species, this study is, to our knowledge, the first to provide a global map of species invasions for a given taxonomic group and should stimulate others to test the generality of these findings for other taxa at this spatial scale. Such broad-scale analyses would help local researches to focus on non-native species control in the most sensitive areas (e.g., the six invasion hotspots we identified here for freshwater fish). This study should also stimulate researches on freshwater ecosystems by combining the existing global scale databases of physical disturbances [44,45] and the global pattern of fish invasions given here. This would permit to quantify river basins threats by considering simultaneously different sources of disturbance. Such an approach is urgently needed as rivers are among the most threatened ecosystems of the world [46] and as freshwater fish constitute a major source of protein for a large part of the world population [46].

## Materials and Methods

### Databases.

We conducted an extensive literature survey of native and non-native freshwater fish species check lists. Only complete species lists at the river basin scale were considered, and we discarded incomplete check lists such as local inventories of a stream reach or based only on a given family. The resulting database was gathered from more than 400 bibliographic sources including published papers, books, and grey literature databases (references available upon request). Our species database contains species occurrence data for the world's freshwater fish fauna at the river basin scale (i.e., 80% of all freshwater species described [47] and 1,055 river basins covering more than 80% of Earth's surface). It constitutes the most comprehensive global database for freshwater fish occurrences at the river basin scale and, to our knowledge, the largest database for a group of invaders. We considered as non-native a species (i) that did not historically occur in a given basin and (ii) that was successfully established, i.e., self-reproducing populations. Estuarine species with no freshwater life stage were not considered in our analyses.

The environmental and human databases contain seven variables selected to test (i) the “human activity” hypothesis: human population density (number of people km^−2^), percentage of urban area and purchase power parity GDP (in US$); (ii) the “biotic acceptance” hypothesis: number of native fish species, basin area (km^2^), altitudinal range (m), net primary productivity (NPP in kg-carbon m^−2^ year^−1^), and (iii) the “biotic resistance hypothesis”: number of native fish species. The area of each river basin was taken from published and unpublished data. The altitudinal range for each river basin was determined from a geographical atlas. We calculated the mean value of NPP, human population density, GDP, and percentage of urban area over the surface area of each basin from 0.5° × 0.5° grid data available in the Center for International Earth Science Information Network (CIESIN) and the Atlas of Biosphere [48,49]. The surface area and altitudinal range at the river basin scale are used as quantitative surrogates for habitat heterogeneity [16], which is known to influence native freshwater fish species richness [15,16]. Net primary productivity is used as a quantitative surrogate to river basin energy availability [16] and strongly correlates to native freshwater fish species richness [15,16]. This is verified in our data, as we found that both basin area and NPP are positively correlated to native species richness (partial Pearson's correlation coefficient: *r* = 0.592 and *p* < 0.0001 for basin area while controlling for the effect of NPP; *r* = 0.514 and *p* < 0.0001 for NPP while controlling for the effect of the basin area). Then, the human population density, percentage of urban area, and GDP were used as quantitative surrogates for propagule pressure and habitat disturbance [5,9,33]. The GDP measures the size of the economy and is defined as the market value of all final goods and services produced within a region in a given period of time.

### Fish invasions mapping.

We first mapped the worldwide distribution of (i) the non-native species richness per basin and (ii) the percentage of non-native species per basin (i.e., the ratio of non-native species richness/total species richness). To do that, each basin was delimited by a geographic information system (GIS) using a grid reference of 0.5° latitude and 0.5° longitude and then reported on a world map. We used three classes of percentage (Figure 1A) and richness (Figure 1B) of non-native species to draw colour maps. Other maps with more classes were tried and provided similar results. We selected the one that minimised differences in sample size (i.e., number of river basins) between classes. The percentage of non-native species per basin was used to define invasion hotspots where more than a quarter of the species are non-native (i.e., the third class of percentage of non-native species; red areas in Figure 1A). It was preferred to the richness in non-natives due to its independence from native richness and basin area. For each of the three levels of fish invasion ([ 0%–5% ], ]5%–25%], ]25%–95% ]), we determined the percentage of species facing a high to extremely high risk of extinction in the wild, i.e., the vulnerable, endangered, and critically endangered fish species according to the IUCN Red List [25]. The percentage of threatened species should be regarded with caution, because the IUCN Red List for freshwater fish is still incomplete. The percentages of threatened species for the three levels of fish invasion are therefore probably underestimated. Although we recognise the potential biases and limitations of the IUCN listing procedure, the IUCN Red List of threatened species remains the most objective and authoritative system for classifying species in terms of the risk of extinction at the global scale [41,50]. The list of basins for the three levels of invasion is provided in Dataset S1.

### Modelling method.

In this study, to test the three hypotheses (i.e., “human activity”, “biotic acceptance”, and “biotic resistance”), we did not build the best single and parsimonious model by using stepwise selection of a subset of independent variables having a significant effect on the number of non-native species per basin (i.e., predictive approach). Indeed, a single best model is not necessarily the best explanatory model, because minimizing the overall difference between the observed and predicted values does not necessarily equate to determining probable influence in a multivariate setting [26–28,51,52]. In addition, a simple regression model cannot identify situations in which potentially important independent variables are suppressed by other variables due to their high colinearity. When there is colinearity between independent variables, the direct response of the dependent variable to a independent variable may in fact only be an indirect effect owing to high dependence of the considered variable with one or many others [27].

In our dataset, the seven environmental and human variables are not independent (Pearson's correlation coefficient values ranging from −0.25 to 0.79, Table S1). We therefore evaluated the independent explanatory power of each environmental and human variable by using hierarchical partitioning [26–28,51,52], a method based on the theorem of hierarchies in which all possible models in a multiple regression setting are considered jointly to attempt to identify the most likely causal factors (explanatory approach).

If we consider *k*, the number of explanatory variables (*X*_1_, *X_i_*,…, *X_k_*), there are 2*^k^* possible models (i.e., 128 submodels by considering the seven explanatory variables), including the null model (*M*_0_). The *R_i_* is a measure of fit between one independent variable *X_i_* and the dependent variable *Y*. The fit between each of the seven explanatory variables and the dependent variable *Y* (number of non-native fish species per basin) was measured by the reduction of deviance generated by introducing a given variable into all of the possible models built with the six other variables within the considered hierarchies. We used a generalised linear model (GLM) with a Poisson error to treat our count data (i.e., the number of non-native fish species per basin). Each explanatory variable was log-transformed to meet the assumptions of normality and homoscedasticity.

We consider *k*! hierarchical orderings of models that always begin with *M*_0_ and end with 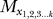 . For any given initial variable *X_i_*, there are (*k* – 1)! possible hierarchies containing *k*(*k* – 1)! models in which *X_i_* appears. For each hierarchy, we evaluate the influence of *X_i_* on each of the *k* models including *X_i_* (increase in model fit generated by including the variable *X_i_* within each model). The independent influence (*I_i_*) of *X_i_* on *Y* was obtained by averaging all of the *k*(*k* – 1)! increases of fit. This averaging alleviates multicolinearity problems that are ignored by using a simple regression model [26–28]. The joint component *J_i_* (effect caused jointly with the *k* – 1 other variables) is obtained by subtracting *I_i_* from *R_i_*, with *R_i_* = *I_i_* + *J_i_*. If all explanatory variables were completely independent of one another, there would be no joint contributions [26]. For each variable, the independent and joint contributions are expressed as the percentage of the total explained deviance (*R*)

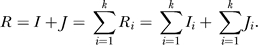



In our models, the total independent contribution accounts for 75% of the total explained deviance, which means that the joint contribution of each explanatory variable was weak in explaining the global variation in non-native species richness (Figure S1). We therefore quantified the independent effect (*IE_i_*) of each variable on the dependent variable *Y* as the percentage of the total independent contribution, i.e. 
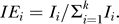
 The significance of the independent effect (*IE_i_*) of each variable was determined by a randomization approach (*n* = 100) which yielded Z-scores [52]. Statistical significance was based on an upper confidence limit of 0.95. Each variable display a significant independent effect.

We applied hierarchical partitioning to a subsample of 597 basins (Afrotropical: 72; Australian: 94; Nearctic: 127; Neotropical: 68; Oriental: 29; Palearctic: 207) for which all seven environmental and human variables used were available. To test potential bias due to differences in sampling effort between continents, hierarchical partitioning was run on 1,000 random subsets of 100 basins among the total of 597 basins. For each variable, we calculated the 95% bootstrap percentile confidence interval of the independent effect (*IE_i_*). Hierarchical partitioning was conducted using the ‘hier.part' package [52] version 1.0–1 implemented on the open source R software [53]. Hierarchical partitioning implemented for linear relationships was relevant to our data, because preliminary analyses did not detected any significant effect of polynomial terms. The hierarchical partitioning results were compared with those obtained with another method (i.e., variation partitioning, [54]). Overall, the results of the two methods were similar, and the variables highlighted as significant by the two approaches were the same.

Hierarchical partitioning does not provide information on the form of the relationship (positive or negative) between the number of non-native species and each explanatory variable. To test the “human activity” hypothesis, we analysed the form and the significance of the relationship between each variable related to the “human activity” hypothesis (GDP, percentage of urban area, and population density) and the residuals from a GLM with a Poisson error. This model explains the number of non-native species by using independent variables related to the “biotic resistance” and “biotic acceptance” hypotheses (number of native species, altitudinal range, basin area, and net primary productivity). This allowed us to control for the effects of environmental conditions and native species richness. Then, to test the “biotic acceptance” hypothesis, we analysed the form and the significance of the relationship between each variable related to the “biotic acceptance” hypothesis (i.e., number of native species, altitudinal range, basin area, and net primary productivity) and the residuals from a GLM explaining the number of non-native species by using the human activity–related variables (i.e., GDP, percentage of urban area, and population density). This allowed us to control for the effects of propagule pressure and habitat disturbance. Lastly, to test the “biotic resistance” hypothesis, we analysed the form and the significance of the relationship between the number of native species and the residuals from a GLM explaining the number of non-native species by using independent variables related to the “biotic acceptance” and “human activity” hypotheses (i.e., altitudinal range, basin area, net primary productivity, GDP, percentage of urban area, and population density). This allowed us to control for the effects of environmental conditions, propagule pressure and habitat disturbance. To test the relationship between the model residuals and each explanatory variable, we performed a Spearman rank correlation test, because the model residuals were not normally distributed.

## Supporting Information

Dataset S1 Names and Invasion Levels of the 1,055 River Basins The three invasion levels are those used in Figure 1A (i.e., the percentage of non-native species per basin). (i) [ 0%–5% ]; (ii) ]5%–25%]; (iii) ]25%–95% ]. Longitude and latitude at the river mouth was also provided for the 1,055 river basins.
(92 KB XLS)

Figure S1 Results from Hierarchical Partitioning Analysis Illustrating the Independent and Joint Contributions of the Explanatory Variables in Accounting for the Variation in Non-Native Species Richness between River Basins (*n* = 597) Values are presented as the percentage of the total explained deviance extracted from a GLM with a Poisson error. The total independent contribution of the explanatory variables accounts for 75% of the total explained deviance.
(46 KB PDF)

Table S1 Pearson's Correlation Coefficient (*r*) between Each Explanatory Variable NSR: native species richness; AR: altitudinal range; BA: basin area; NPP: net primary productivity; GDP: gross domestic product; PUA: percentage of urban area; PD: population density. Bold values indicate a significant correlation *p* < 0.002 (Bonferroni correction, α = 0.002).
(52 KB PDF)

## Abbreviations

GDP: gross domestic product
GLM: generalised linear model
IUCN: The World Conservation Union
NPP: net primary productivity
