# Supplementary material for: Fish Invasions in the World's River Systems: When Natural Processes Are Blurred by Human Activities
Source: PLoS Biol. 2008 Feb 5;6(2):e28. doi: 10.1371/journal.pbio.0060028 (PMC2225436; doi:10.1371/journal.pbio.0060028)
Supplement: Supplementary file 1 [file pbio.0060028.sg001.pdf]

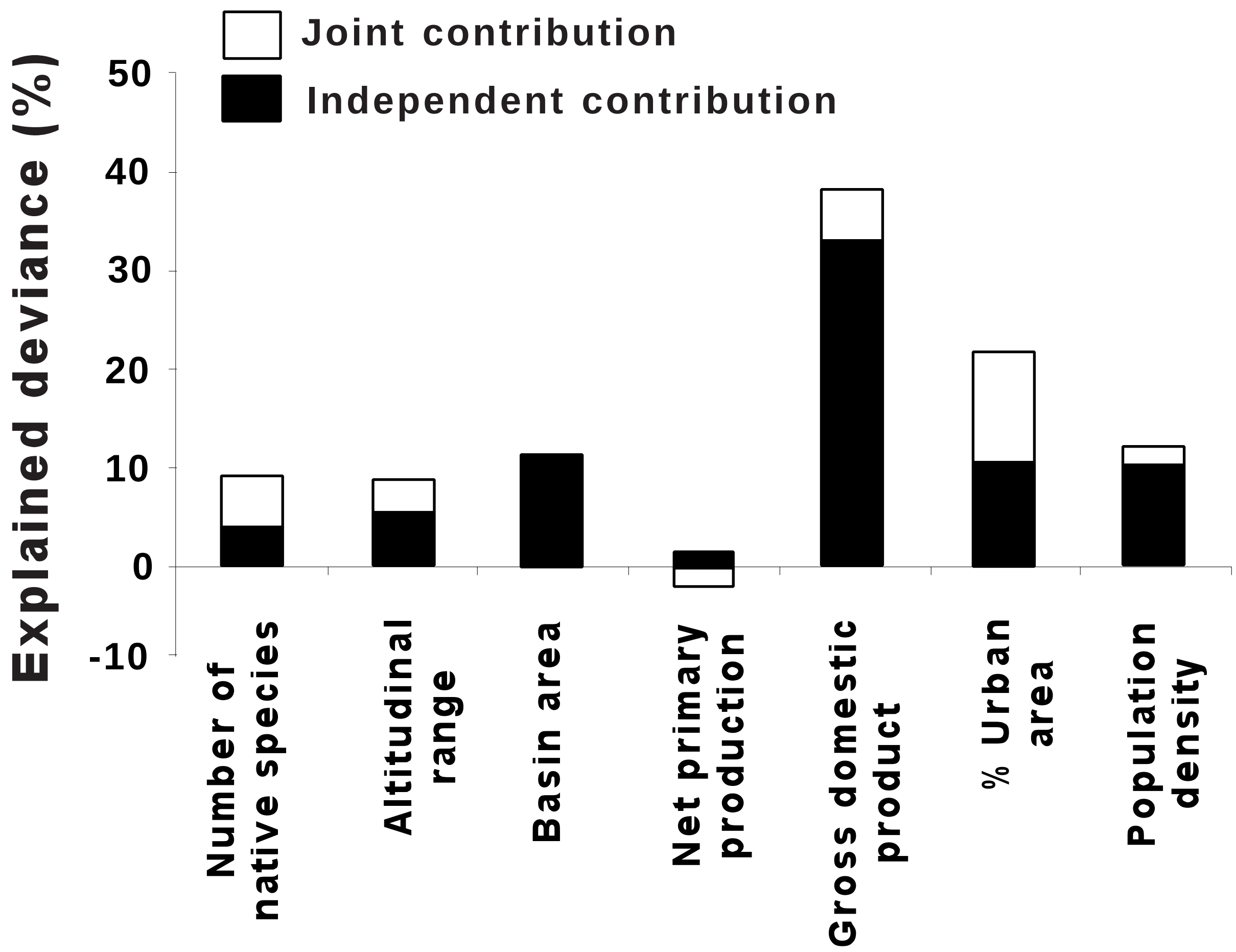

Figure S1: Results from hierarchical partitioning analysis illustrating the independent and joint contributions of the explanatory variables in accounting for the variation in non-native species richness between river basins (n=597). Values are presented as the percentage of the total explained deviance extracted from a generalized linear model with a Poisson error. The total independent contribution of the explanatory variables accounts for 75% of the total explained deviance.
