# Supplementary material for: Fish Invasions in the World's River Systems: When Natural Processes Are Blurred by Human Activities
Source: PLoS Biol. 2008 Feb 5;6(2):e28. doi: 10.1371/journal.pbio.0060028 (PMC2225436; doi:10.1371/journal.pbio.0060028)
Supplement: Supplementary file 2 [file pbio.0060028.st001.pdf]

Table S1: Pearson correlation coefficient (r) between each explanatory variable (NSR: number of natives species; AR: altitudinal range; BA: basin area; NPP: net primary productivity; GDP: gross domestic product; PUA: percentage of urban area, PD: population density). Bold values indicate a significant correlation, p<0.002 (bonferroni correction, α=0.002).

	NSR	AR	BA	NPP	GDP	PUA	PD
NSR	1.00						
AR	**0.17**	1.00					
BA	**0.47**	**0.31**	1.00				
NPP	**0.33**	0.01	**-0.25**	1.00			
GDP	**0.16**	0.10	**-0.15**	**0.30**	1.00		
PUA	**0.14**	-0.10	**-0.16**	**0.18**	**0.64**	1.00	
PD	**0.15**	0.08	**-0.19**	**0.34**	**0.79**	**0.60**	1.00
